# How often is the office visit needed? Predicting total knee arthroplasty revision risk using pain/function scores

**DOI:** 10.1186/s12913-016-1669-y

**Published:** 2016-08-24

**Authors:** Charles D. Hightower, Lisa S. Hightower, Penny J. Tatman, Patrick M. Morgan, Terence Gioe, Jasvinder A. Singh

**Affiliations:** 1 Alaska Native Medical Center, Anchorage, AK USA; 2 Department of Agricultural and Extension Education, Virginia Tech, Blacksburg, VA USA; 3 Health East Education and Research Department, St. Paul, MN USA; 4 Department of Orthopaedic Surgery, University of MN Medical School, Minneapolis, MN USA; 5 Department of Orthopaedic Surgery, Minneapolis VAMC, Minneapolis, MN USA; 6 Birmingham VAMC and University of Alabama at Birmingham, Faculty Office Tower 805B, 510 20th Street S, Birmingham, AL 35294 USA

**Keywords:** Total knee arthroplasty, TKA, Revision risk, Pain, Function, American Knee Society Scores, AKS scores

## Abstract

**Background:**

Most patients have favorable outcomes after primary total knee arthroplasty (TKA). Well-validated methods to predict the risk of poor outcomes have not been developed or implemented. Several patients have annual clinic visits despite well-funcitoning TKA, as a routine practice, to detect early failure requiring revision surgery. It is not known whether assessment of pain and function can be used as a predictive tool for early failure and revision to guide practice. Our objective was to determine whether pain and function can predict revision after TKA.

**Methods:**

We retrospectively studied data from a large prospectively gathered TKA registry to examine changes in outcome scores for primary TKAs undergoing revision compared to those not requiring revision to determine the factors that are predictive for revision.

**Results:**

Of the 1,012 patients, 721 had had a single-sided primary TKA and had American Knee Society (AKS) Scores for three or more visits. 46 patients underwent revision, 23 acutely (fracture, traumatic component failure or acute infection) and 23 for latent causes (late implant loosening, progressive osteolysis, or pain and indolent infection). Mean age was 70 years for the non-revision patients, and 64 years for those revised. Both AKS Clinical and AKS Function Scores for non-revised patients were higher than in revision patients, higher in acute revision compared to latent revision patients. Significant predictors of revision surgery were preoperative, 3- and 15-month postoperative AKS Clinical Scores and 3-month AKS Function Scores. At 15-month post-TKA, a patient with a low calculated probability of revision, 32 % or less, was unlikely to require revision surgery with a negative predictive value of 99 %.

**Conclusion:**

Time dependent interval evaluation post-TKA with the AKS outcome scores may provide the ability to assign risk of revision to patients at the 15-month follow-up visit. If these findings can be replicated using a patient-reported measure, a virtual follow-up with patient-reported outcomes and X-ray review may be an alternative to clinic visit for patients doing well.

**Electronic supplementary material:**

The online version of this article (doi:10.1186/s12913-016-1669-y) contains supplementary material, which is available to authorized users.

## Background

The demand for total joint arthroplasty is expected to increase significantly by the year 2020 in the US and other countries [1]. Such volume will invariably increase the need for both surgical resources and the resources required to follow these patients over time. Survivorship for modern total knee arthroplasty (TKA) has generally been reported at 15 years or more with an estimated incidence of failure of 1 % per year [1–3]. Although the most appropriate protocol for following the TKA patient has not been established, an American Association of Hip and Knee Surgeons (AAHKS) survey advocates biannual or annual clinic follow-up visits even for asymptomatic patients after primary TKA [4]. These visits routinely include a radiographic evaluation and an associated physical exam by an orthopaedic surgeon or qualified provider. One major motivation for longitudinal surveillance is the early identification of clinically significant osteolysis in the asymptomatic patient.

Total knee failures for fracture, component failure or acute infection tend to be painful and typically motivate patients to seek prompt clinical evaluation. The latent causes of revision including osteolysis or pain are more insidious and require surveillance by the clinician. Timely intervention for osteolysis has been shown to reduce the overall cost and length of recovery after surgery [5]. Although several studies have used logistic regression modeling to predict variables that impact outcome after arthroplasty surgery [6–9], to our knowledge no study has generated a predictive equation for determining the probability of revision using only outcome scores.

The use of outcome measures in arthroplasty research has focused on investigating the epidemiology of arthroplasty patients, identifying patient risk factors leading to poor clinical outcomes, determining implant survival and quantifying patient experience and satisfaction. One example of such an outcome instrument is the American Knee Society (AKS) Clinical Rating System [10].

Understanding the long-term results of joint arthroplasty can help surgeons improve both their ability to make the correct decision regarding the need for surgery as well as the technical competence required for a successful procedure [11]. Can outcome measurements be used as a predictive tool to guide our surveillance practices? Are there specific time intervals after surgery that predict the need for revision surgery? Can a population-specific equation be generated to calculate probability for revision surgery?

The purpose of this study was to assess the relationship between chronological changes in AKS scores with an end point of revision surgery. We hypothesized that patients with a total knee arthroplasty can be categorized as one of the following: 1) the acutely failed TKA, 2) the TKA at risk of latent failure, or 3) the well-functioning TKA. Furthermore, we hypothesize that these populations can be distinguished by changes in AKS scores over time, independent of information gained from the physician office visit. The following research questions were investigated in this study for a patient population with TKA:

1. On average, at what point did the patients undergo revision surgery after the primary surgery?
2. Do patients with or without revision have statistically different AKS Clinical scores over time?
3. Do patients with or without revision have statistically different AKS Function scores over time?
4. What independent variables are statistically significant predictors of whether patients will undergo revision surgery?

## Methods

One thousand and twelve consecutive patients who underwent TKA at the Minneapolis Veteran’s Affairs (VA) Arthroplasty TKA registry were retrospectively reviewed from 1993 to 2008. Of the 1012 patients in the database, 721 patients met all of the following inclusion criteria for analysis: 1) the American Knee Society (AKS) Scores were available for three or more visits, 2) the AKS Scores fit within the appropriate 0 to 100 scale, and 3) patients with a single-sided primary total knee arthroplasty. The sample of 721 patients included 46 patients who ultimately underwent revision arthroplasty and 675 non-revision patients. The average age of all of the patients in the study (*n* = 721) was 69 years. The average age of the non-revision patients (*n* = 675) was 70 years, and the average age of the patients who underwent revision TKA (*n* = 46) was 64 years. Twenty-three knees were revised for fracture, traumatic component failure or acute infection. These were categorized as acute causes for revision. The remaining 23 knees were revised for implant loosening, progressive osteolysis, or pain and indolent infection categorized as latent causes for revision. AKS scores were assessed from the preoperative visit and subsequent postoperative follow-up visits.

Data were collected on the 721 patients including age at primary surgery, AKS Clinical Scores, and AKS Function Scores. For the 46 revision patients, the time interval in months between the primary surgery and revision surgery was also collected. Visits were scheduled at regular intervals for all patients, but were altered as needed to accommodate patient needs. In order to perform logistic regression modeling focusing on changes in the AKS scores, time intervals were normalized for patients at 3-month time intervals from 0 months (preoperative visit) until 48 months. We considered but decided not to have smaller intervals than 3-months, since regular pain/function assessments are typically not done any more frequently than 3-monthly. All patients had a minimal follow-up of 12-months.

The AKS is a disease-specific, provider-administered outcome instrument developed in 1989. Designed as a dual rating system-- evaluating the knee arthroplasty separate from the patient’s functional status [10]-- the developers of the AKS sought to isolate arthroplasty-specific changes from those global changes in a patient’s health and function attributable to aging. AKS Clinical scores are generated from 100 points possible with deductions for pain, malalignment, and limited ROM. Importantly, a high AKS Clinical score reflects less pain. The AKS Functional score is a 100 point index with deductions for limitation in walking and use of stairs, and for the use of assistive devices and/or railings. When compared to the Western Ontario Mcmaster osteoarthritis Index (WOMAC) and Short-Form-36 (SF-36), the AKS demonstrates acceptable construct validity, reliability and responsiveness [12, 13]. Normative values for the AKS in the elderly have been established for general control comparison [14]. The AKS provides information that is non-specific for unilateral versus bilateral total knee arthroplasty. Although the AKS Score was recently extensively revised, tested, and released for use in early 2012, most arthroplasty surgeons have had more experience with the earlier version described.

Missing data points were calculated using the one of the following rules: 1) existing data was entered if the visit occurred within one month of the 3-month time interval, 2) values were imputed if one time interval was missing between two intervals where existing data was present and had the same values (<20 % were imputed), 3) values were calculated using individual patient regression model estimates for revision patients, and 4) values were calculated following the trends for the individual patient’s scores.

Individual patient regression models were used to calculate missing interval data for revision patients because the data had curvilinear and linear trends. Since data on average tended to be flat in patients without revision, regression models were not used. After the data was filled in using the first two rules, the regression model data were entered for the patients with revision. The regression estimates needed to fit within the range of data that were entered for the surrounding two time intervals or it was not used.

The final method used to calculate interval data was to use the average of the scores that surrounded the missing data point. For example, if the AKS Clinical Scores were available for 9 months at 50 and 15 months at 70, then the estimate for 12 months would be 60. Revision patients’ data included intervals after revision to complete the 48-month period.

Statistical tests were conducted using SPSS v20. Trend analyses were conducted for the AKS Function Scores and Clinical Scores of the patients over time. Comparisons of the means across different groups of patients were also conducted using analysis of variance (ANOVA) tests. Post-hoc analysis was performed with the Student-Newman-Keuls test. In addition, a stepwise hierarchical logistic regression was used to identify the statistically significant predictors of the probability of revision surgery. This generated an equation to determine the probability of revision TKA. Patients were classified as high or low risk for revision based on their calculated probability for revision TKA.

## Results

### Average time of revision surgery in patients with TKA

**Fig. 1:**
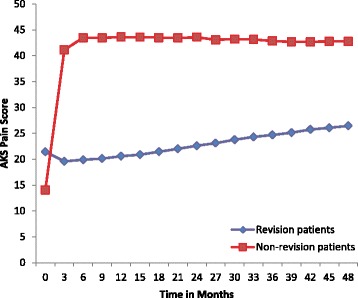
AKS Clinical Score over time for patients with revisions (*n* = 46) and patients without revisions (*n* = 675). ANOVA showed no difference in the AKS Clinical scores between groups at 0 months (Student-Newman-Keuls *P* > 0.05). There was no difference in AKS Clinical scores between the latent and acute groups at 3–18 months, but the non-revision group differed significantly from both the latent and acute groups at these time intervals (*p* = 0.05)

**Fig. 2:**
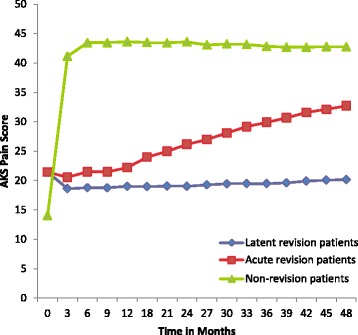
AKS Clinical Score over time for patients with acute revisions (*n* = 23), patients with latent revisions (*n* = 23), and patients without revisions (*n* = 675). All 3 groups had significantly different AKS Clinical scores at the 21–48 month time intervals (*p* = 0.05)

**Fig. 3:**
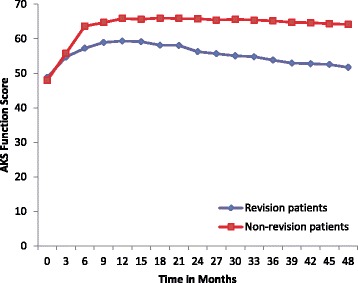
AKS Function Score over time for patients with revisions (*n* = 46) and patients without revisions (*n* = 675). ANOVA showed that there was no difference in the AKS Function scores for any of the 3 groups at 0–21 months (*p* =0.83). From 24–48 months, the acute and latent revision group were not statistically different (Student-Newman-Keuls *p* >0.05)

**Fig. 4:**
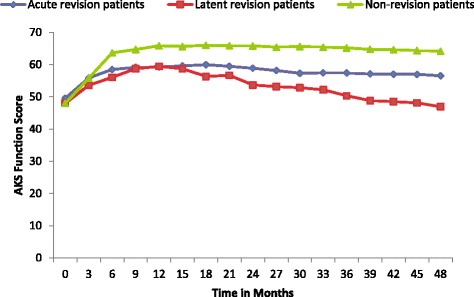
AKS Function Scores for patients with acute revisions (*n* = 23), patients with latent revisions (*n* = 23), and patients without revisions (*n* = 675). From 24–48 months, the acute revision and non-revision group were also statistically similar. The latent group and the non-revision group were statistically different (Student-Newman-Keuls *p* = 0.05)

The average time between primary surgery and revision surgery is 3.1 years (range = 0.4 years to 11.8 years). Approximately 19.6 % of patients had revision surgery less than a year after their primary surgery, 54.3 % of patients had revision surgery between 1–4 years after primary surgery, and 26.1 % of patients had revision surgery more than 4 years after their primary surgery. A range of 0–4 years accounts for 74 % of all revisions surgeries. The figures show the association of AKS clinical score with overall revision risk (Fig. 1) and the risk of revision type, early vs. latent vs. none (Fig. 2) and of AKS function score with overall revision (Fig. 3) and revision type (Fig. 4).

### Statistically significant predictors of the probability of revision surgery

Binary logistic regression analyses were conducted to determine the statistically significant predictors of the probability of revision surgery. Variables included 1) age of the patients, 2) AKS Clinical Scores 3) AKS Function Scores. Multiple regression models were generated to determine the optimal model to predict the probability of having revision surgery with the most clinical applicability. Clinical applicability was targeted at a model with the highest predictive value using variables closest to the time of primary surgery. The following four independent variables were found to be significant predictors of revision: AKS Pain Score at 0 months (*p*. < 0.0001), AKS Clinical Score at 3 months (*p* < 0.0001), AKS Clinical Score at 15 months (*p* < 0.0001), and AKS Function Score at 3 months (*p* = 0.004, Table 1).

**Table 1:** Statistically significant variables that predict the probability of having revision surgery

Independent variables	Wald test	Significance level^a^	Coefficient (B)
AKS Clinical Score at 0 months	24.616	0.000	0.113
AKS Clinical Score at 3 months	36.301	0.000	−0.156
AKS Clinical Score at 15 months	35.109	0.000	−0.107
AKS Function Score at 3 months	8.369	0.004	0.043

^a^The significance level for this study has been set at *p*-values equal to or less than 0.05

The optimal logistic regression equation model for our TKA Registry is:

$$ \begin{array}{l}\mathbf{Log}\ \mathbf{o}\mathbf{dd}{\mathbf{s}}_{\mathbf{Revision}\ \mathbf{S}\mathbf{urgery}} = \mathbf{0}.\mathbf{703} + \mathbf{0}.\mathbf{113}\ \left(\mathbf{A}\mathbf{K}\mathbf{S}\ \mathbf{Clinical}\ \mathbf{S}\mathbf{cores}\ \mathbf{f}\mathbf{o}\mathbf{r}\ \mathbf{0}\ \mathbf{months}\right)\ \\ {}\hbox{--}\ \mathbf{0}.\mathbf{156}\ \left(\mathbf{A}\mathbf{K}\mathbf{S}\ \mathbf{Clinical}\ \mathbf{S}\mathbf{cores}\ \mathbf{at}\ \mathbf{3}\ \mathbf{months}\right)\ \\ {}\hbox{--}\ \mathbf{0}.\mathbf{107}\ \left(\mathbf{A}\mathbf{K}\mathbf{S}\ \mathbf{Clinical}\ \mathbf{S}\mathbf{cores}\ \mathbf{at}\ \mathbf{15}\ \mathbf{months}\right) + \mathbf{0}.\mathbf{043}\ \left(\mathbf{A}\mathbf{K}\mathbf{S}\ \mathbf{Function}\ \mathbf{S}\mathbf{cores}\ \mathbf{at}\ \mathbf{3}\ \mathbf{months}\right)\end{array} $$

When applied to the revision group, the average calculated probability from this equation is 59 % (standard deviation [SD] +/- 20 %). The average probability is 4 % (SD +/- 14 %) for the non-revision group. Two standard deviations above the patient without revisions mean would include a range from 0–32 %. The range for high risk for revision surgery was selected as 32 % or greater. Using this range, patients requiring revision surgery are predicted with a sensitivity of 89 % and specificity of 96 % (Additional file 1). The positive predictive value of this range is 63 %. The negative predictive value for patients identified as low risk to not undergo surgery is 99 % (Additional file 1). If a patient’s calculated probability for revision is less than 32 % at the 15 month visit, they are highly unlikely to require revision surgery by the 4 years post-operative visit. Examples of the use of the prediction equation are shown in Additional file 1.

## Discussion

As the demand for primary total joint arthroplasties continues to increase, alternate means of longitudinal follow-up will be necessary. What information should be collected from our patients, and at what time interval remains a topic of discussion. In this retrospective review of 1,012 total knee arthroplasties we found that the probability of requiring revision surgery could be calculated using AKS data from 3 critical postoperative time intervals: 0, 3, and 15 months. This model can account for 67 % of the variance concerning the probability of the patients needing revision surgery. This model effectively discriminated between revision and non-revision patients. The age of the patient at the time of revision and the length of time between primary surgery and revision were not significant predictors for revision surgery. Our findings provide further support to previous findings that Oxford knee and hip scores at 6-months and Oxford hip scores at 5-years were predictive of subsequent early revision [15, 16]. A recent UK study showed that review of questionnaire and radiograph together (but not either alone) identified all patients in need of increased surveillance after TKA/THA [17]. Our findings of association of pain/function scores with revision risk are similar to these previous studies. An advance from our study is the development of a prediction model using scores from validated questionnaires.

The proposed prediction equation would require AKS to be measured preoperatively and at 3 and 15 months post-TKA (at least two office visits). Some providers may only be following their patients on an as needed basis after the initial wound check in the first 2–6 weeks post-TKA. Patient-reported outcome measures may be able to capture data that were captured in AKS scores (a physician-administered measure), and may be a more practical alternative to AKS. Our prediction equation needs to be replicated using patient-reported outcome measures.

Although consensus recommendations in the US have favored annual or biannual follow-up [4], this has not been globally accepted. Bankes and colleagues [18] reviewed 1000 questionnaires to the British Orthopaedic Association (BOA) concerning follow-up practices. They found that 50 % of surgeons followed their total hip patients less than 1 year. The majority of providers (78 %) followed them for under 5 years, and only 14 % had indefinite follow-up. The authors reported that cost was a major deterrent of annual follow-up for all arthroplasty patients [18]. In our population, 73.9 % of revisions occurred early and were captured within the first 4 years.

The utility of the physician visit for well functioning arthroplasties has been questioned. Bhatia and Obadare [19] performed a cost-benefit analysis of 100 consecutive patients and found that the 304 visits with radiographs for 100 patients over a 2 year period resulted in a cost of £23,397 ($38,970 in 2003 USD). There were 10 patients that had issues requiring interventions, of which three were found in clinic follow-ups and seven identified by General Practice referral or in the Emergency Department. They noted that most issues that required intervention were found at the first postoperative visit. Their recommendations were for routine follow-up for 6–12 weeks [19]. The BOA recommends AP/lateral radiographs at 5 years and every 5 years thereafter for long-term surveillance (*Anon. Total Hip Replacement: A Guide to Best Practice 1999*). The British National Health Service also notes the waiting list time for new arthroplasty patient evaluation as a significant consideration for resource utilization [18, 19].

Patient experience and satisfaction have been driving forces in US healthcare reform and optimization. Sethuraman surveyed 100 patients during routine arthroplasty follow-up about their preference for office visits [20]. A significant proportion (45 %) would have preferred not to have an office visit, citing wages lost and potential time spared as determining factors. None of these patients felt that the quality of their care would be jeopardized by not having an office visit. It was noted that the remaining 55 % noted that office visits and routinely seeing their surgeon was important for maintaining quality of care and satisfaction. The authors recommended asking patients their preference and following those not interested in office follow-up with radiographs and completion of an outcomes measure.

There are limitations to the conclusions that can be drawn from the current study. The ability to generalize results using our population’s logistical regression equation is limited to use in similar patient populations with unilateral total knee arthroplasty. Although women represent 66.2 % of the primary total knee arthroplasty patients receiving a joint in one year [21], they represent 1 % of this Veterans Administration arthroplasty patient population. Single-site study and a low number of revisions limit the generalizability. Another limitation is the outcome instrument utilized. This version of the Knee Society Score itself is not without limitations. It is non-specific in its reporting of clinical change and function for patients with bilateral arthroplasties. Lingard and colleagues evaluated the validity and reliability of the AKS compared to the WOMAC and SF-36. They found that although the AKS had adequate convergent construct validity, it had weaker responsiveness and poor inter-item correlation compared with either of the other measures [12]. Both the clinical and function scores reach their maximum improvement at 2-years, followed by subsequent decline as a function of age and as the patient’s number of comorbidities increase [22]. Konig and colleagues prospectively evaluated 276 TKA patients and found that after clinical scores and functional scores plateaued, they tended to decrease by 5 points/year after 2 years postoperative [22]. A notable weakness of this study is the need to normalize the time intervals for generating the regression models required to compare the revision patients’ AKS scores. We realize that standard clinic follow up visits likely occurs at 2 weeks, 6 weeks, 3 months, 6 months and 1 year after the arthroplasty. In order to optimize comparisons of the trends of each individual despite variability of follow-up intervals, we utilized rules to calculate intervening scores. The validity of our conclusions are clearly related to the assumptions made.

Our study predicts the risk of revision only, recognizing that interventions other than revision may be needed by some patients post-TKA. Future studies should examine non-revision interventions as well.

Although this study can act as a basis  for future comparisons, validation of the prediction equation could occur by repeating this study with an increased number of visits at 3 month intervals for 48 months. This may be costly and impractical. Validation could also take the form of replicating the techniques used here to evaluate similar TKA registries that have collected AKS scores over time.

Further research is needed to evaluate other arthroplasty outcome instruments for their ability to predict revision at associated critical time intervals. It may also be important to determine if regression modeling can assist in risk stratification for patients *following* revision surgery, identifying risk for further additional surgical interventions. Interestingly, the preoperative AKS scores did vary significantly between revision and non-revision groups. Previous studies have shown that preoperative differences in depression, pain and anxiety, and gender are predictive of poor outcome following total knee arthroplasty [23–25]. Future research can be directed at quantifying the utility of using preoperative AKS scores to identify patients at risk for poor outcome prior to surgery. Latent and acute revision subgroups were both statistically different from each other, and from the non-revision group. Due to the low number of patients in each of these subgroups it was impossible to create logistical regression equations to predict membership in each of these groups. As we continue to follow our cohort and identify more members in these groups, further analysis can be performed to describe predictive implications.

## Conclusions

In conclusion, we noted that patients with and without revisions have AKS scores that vary significantly over time. Specifically, patients with revisions can be viewed in terms of their mechanism of failure, and patients with latent and acute revisions have statistically different AKS score profiles. The change in AKS scores at set time intervals can be used to calculate the risk of revision vs. non-revision. Risk stratification can provide an objective guide for surveillance practices. Our data suggest that using the AKS Score gathered preoperatively, at 3 months and 15 months postoperatively can aid in understanding an individual patient’s probability for revision. This may provide a powerful incentive for surgeons to participate in arthroplasty registries which include gathering such outcome measurements. Follow-up care guided by risk stratification offers a potential reduction in resource expenditure for the patient, the surgeon, and the medical system.

## Additional file

Additional file 1: Appendix 1. Tabulation of patients who did and did not have revision surgery and thier risk of revision. This supplementary file shows the cross-tabulation of the patients determined to be high- or low-risk based on the equation, who underwent revision surgery. Appendix 2: Interpretation using the Equation with examples. This supplementary file shows two examples of risk of revision in two hypothetical patents with regards to the risk of revision surgery and the proposed clinkical surveillance frequency based on the risk of revision. (DOC 80 kb)

## Abbreviations

AAHKS: American Association of Hip and Knee Surgeons
AKS: American Knee Society
BOA: Bristish Orthpaedic Association
SD: Standard deviation
SF-36: Short-Form-36
TKA: Total knee arthropalsty
WOMAC: Western Ontario Mcmaster osteoarthritis Index
