# Supplementary material for: How often is the office visit needed? Predicting total knee arthroplasty revision risk using pain/function scores
Source: BMC Health Serv Res. 2016 Aug 24;16(1):429. doi: 10.1186/s12913-016-1669-y (PMC4995795; doi:10.1186/s12913-016-1669-y)
Supplement: Additional file 1: Appendix 1. — Tabulation of patients who did and did not have revision surgery and thier risk of revision. This supplementary file shows the cross-tabulation of the patients determined to be high- or low-risk based on the equation, who underwent revision surgery. Appendix 2: Interpretation using the Equation with examples. This supplementary file shows two examples of risk of revision in two hypothetical patents with regards to the risk of revision surgery and the proposed clinkical surveillance frequency based on the risk of revision. (DOC 80 kb) [file 12913_2016_1669_MOESM1_ESM.doc]

**Appendix 1: Tabulation of patients who did and did not have revision surgery and thier risk of revision**

|  | Test Range | |  |
| --- | --- | --- | --- |
| Revision | High risk | Low Risk | Total |
| Yes | 41 | 5 | 46 |
| No | 25 | 650 | 675 |
| total | 66 | 655 | 721 |

High risk = probability >32%, Low risk = Probability <32%

Sensitivity: 89% Specificity: 96%

Positive Predicted Value: 62%

Negative Predictive Value: 99%


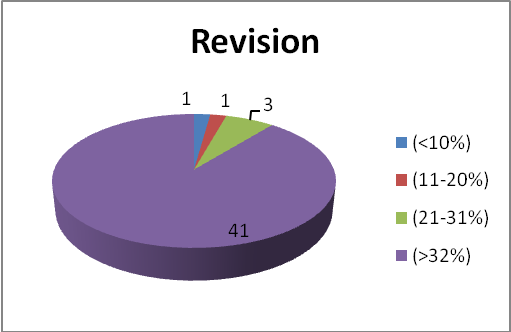


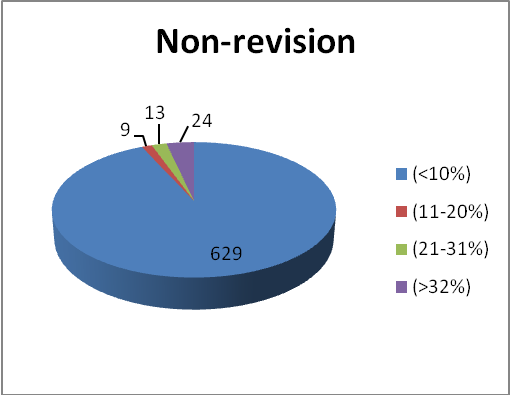


**Appendix 2: Interpretation using the Equation with examples**

In order to provide a better understanding of the application of the predictive logistic equation, two examples are provided below.

*Example 1:* One patient has completed a preoperative visit, a follow-up visit at 3 months, and again at 15 months after the primary surgery. Her AKS Clinical Score for her preoperative visit was 17. At her 3-month follow-up visit her AKS Clinical Score was 17, and her AKS Function Score was 50. At her 15-month follow-up visit her AKS Clinical Score was 24.

The probability that this patient will need revision surgery can be calculated with the following equation:

The log odds of needing revision surgery = 0.703 + 0.113(17) – 0.156(17) – 0.107(24) + 0.043(50)

= 0.703 + 1.921 – 2.652 – 2.568 + 2.15 = - 0.446

The odds of needing revision surgery = e ^-0.446^ = 0.640183772

Therefore the probability of needing revision surgery = 0.640183772 / (1 + 0.640183772)

= .3903122 or 39.03%

**As of her 15 month visit, this patient has a *39.03%* probability of needing revision surgery. This patient would be categorized as high risk for revision surgery (>32%) and should undergo closer surveillance.**

*Example 2:* One patient has completed a preoperative visit, a follow-up visit at 3 months, and again at 15 months after the primary surgery. The AKS Clinical Score for his preoperative visit was 45. At the 3-month follow-up visit his AKS Clinical Score was 50, and his AKS Function Score was 100. At the 15-month follow-up visit his AKS Clinical Score was 45.

The probability that this patient will need revision surgery can be calculated with the following equation:

The log odds of needing revision surgery = 0.703 + 0.113(45) – 0.156(50) – 0.107(45) + 0.043(100)

= 0.703 + 5.09 – 7.8 – 4.815 + 4.3 = -2.522

The odds of needing revision surgery = e ^-2.522^ = 0.08

Therefore the probability of needing revision surgery = 0.08 / (1 + 0.08)

= .07407 or 7%

**As of his 15 month visit, this patient has a *7%* probability of needing revision surgery. This patient would be categorized as low risk for revision (<32%) and may benefit from less frequent clinical surveillance.**
